# Multifaceted aging and rapamycin

**DOI:** 10.18632/aging.100575

**Published:** 2013-07-11

**Authors:** Vladimir N. Anisimov

**Affiliations:** Department of Carcinogenesis and Oncogerontology, N.N. Petrov Research Institute of Oncology, St.Petersburg, 197758 Russia

Aging is commonly defined as a time-dependent loss of physiological integrity, leading to the decline and impair in organism functions and to the increase of risk for cancer and other major age-associated diseases, finally increasing vulnerability to death [1]. During the last decade the intensive search of anti-aging remedies has lead to the conclusion that both the insulin/IGF-like signaling (IIS) and nutrient response pathways such as the mechanistic target of rapamycin (MTOR) control aging and age-associated pathology in yeast, worms, insects and mammals [2-6]. mTOR complex 1 (mTORC1) is activated by insulin and related growth factors through phosphatidylinositol-3-OH kinase (PI(3)K) and AKT kinase signaling and suppressed by AMP-activated protein kinase (AMPK), a key sensor of cellular energy status. mTORC1 involved into promotion messenger RNA translation and protein synthesis through ribosomal protein S6 kinases (S6Ks) and 4E-BP protein, which in the hypophosphorylated form acts as a negative regulator of the cap-binding protein eIF4E. mTORC1 also stimulates lipid biosynthesis, inhibits autophagy, and through hypoxic response transcription factor HIF-1α regulates mitochondrial function and glucose metabolism. Rapamycin suppresses mTORC1 and also indirectly mTORC2 that leads glucose intolerance and abnormal lipid profile. Effects of biguanides and rapamycin on the senescence-associates secretory phenotype interfering with IKK-β/NF-κB – an important step in hypothalamic programming of systemic aging. Recent finding of suppressive effect of rapamycin on some parameters of brain aging in mice [7] and in senescence-accelerated OXYS rats [8] have shown that the drug controls multiple events related to aging. There are nine tentative hallmarks of aging in mammals, which may represent common denominators of aging in different organisms: genomic instability, telomere attrition, epigenetic alterations, loss of proteostasis, deregulated nutrient sensing, mitochondrial dysfunction, cellular senescence, stem cell exhaustion, and altered cell-to-cell communication [1]. Rapamycin and metformin seem to influence all of them. Noteworthy, there is a significant similarity in the effects of rapamycin and metformin as anti-aging and anti-carcinogenic remedies. We believe that rapamycin and metformin are promising for premature prevention in humans.
